# Supplementary material for: Emergent constraints on carbon budgets as a function of global warming
Source: Nat Commun. 2024 Feb 29;15:1885. doi: 10.1038/s41467-024-46137-7 (PMC10904375; doi:10.1038/s41467-024-46137-7)
Supplement: Supplementary file 1 — Supplementary Information [file 41467_2024_46137_MOESM1_ESM.pdf]

Emergent Constraints on Carbon Budgets for the Paris Climate Targets

Peter M. Cox[1,2], Mark S. Williamson[1,2], Pierre Friedlingstein[1,2], Chris D. Jones[3], Nina M.Raoult[1,2], Joeri Rogelj[4] and Rebecca M. Varney[1,2]

[1]*Faculty of Environment, Science, and Economy, University of Exeter, UK*
[2]*Global Systems Institute, University of Exeter, UK*
[3]*Met Office-Hadley Centre, Fitzroy Road, Exeter, UK*
[4]*Centre for Environmental Policy and Grantham Institute, Imperial College London, London, UK*

Supplementary Information

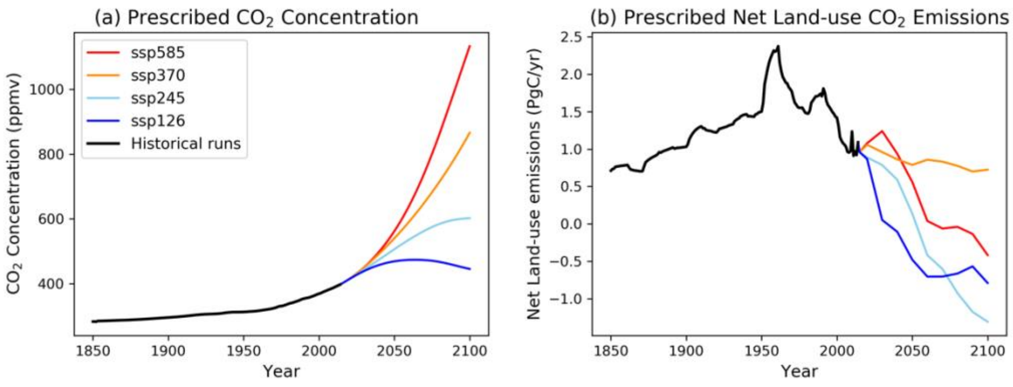

Figure S1: Prescribed inputs to the CMIP6 ESM runs: (a) global mean atmospheric CO_2 concentration; (b) net carbon emissions from land-use change. Black lines show the prescribed historical data (1850-2014) and coloured lines represent each of the four ssp scenarios (2015-2100).

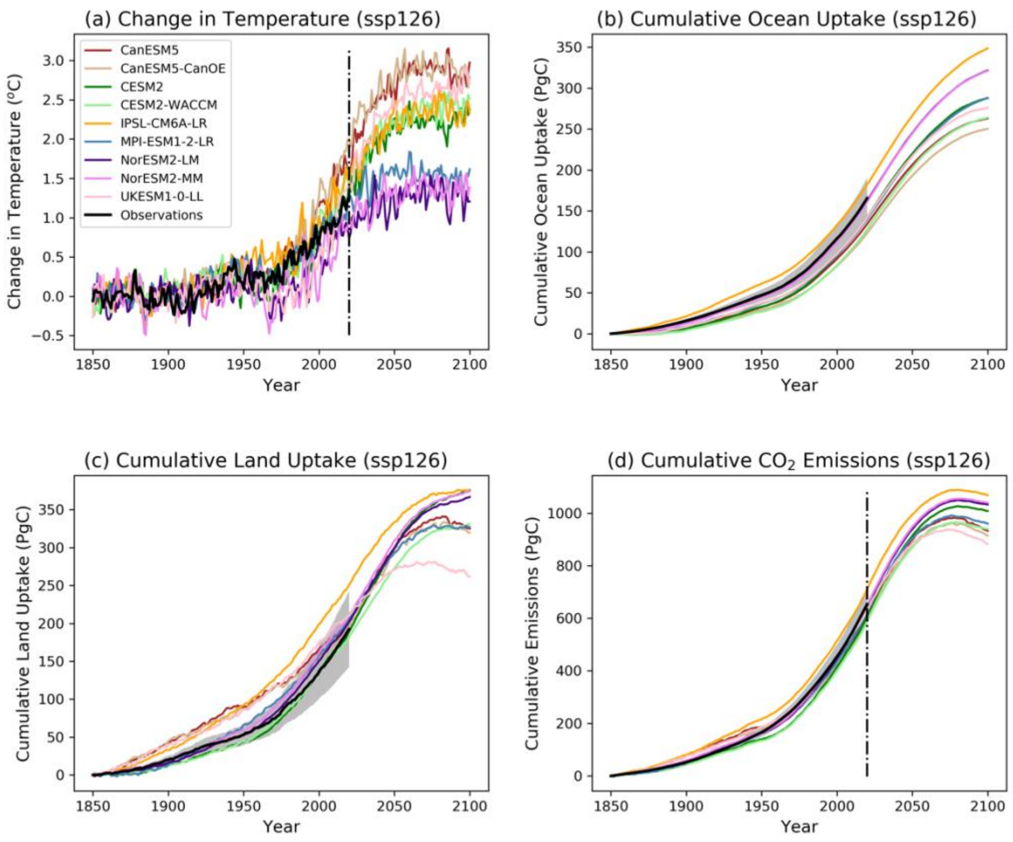

Figure S2: As figure 1 but for the SSP126 scenario, annual global mean anomalies relative to the 1850-1899 mean: (a) temperature change; (b) cumulative ocean carbon uptake; (c) cumulative land carbon uptake; (d) implied cumulative emissions (diagnosed using equation 1). Each coloured line represents a different CMIP6 Earth System Model (ESM) as identified in the key on panel (a). The thick black line in panel (a) is the observational estimate from IPCC AR6 WG1 Chapter 2. The thick black lines in panels (b)-(d) represent observational estimates from the Global Carbon Project, with grey bands showing the estimated standard error in these values (see Methods for details).

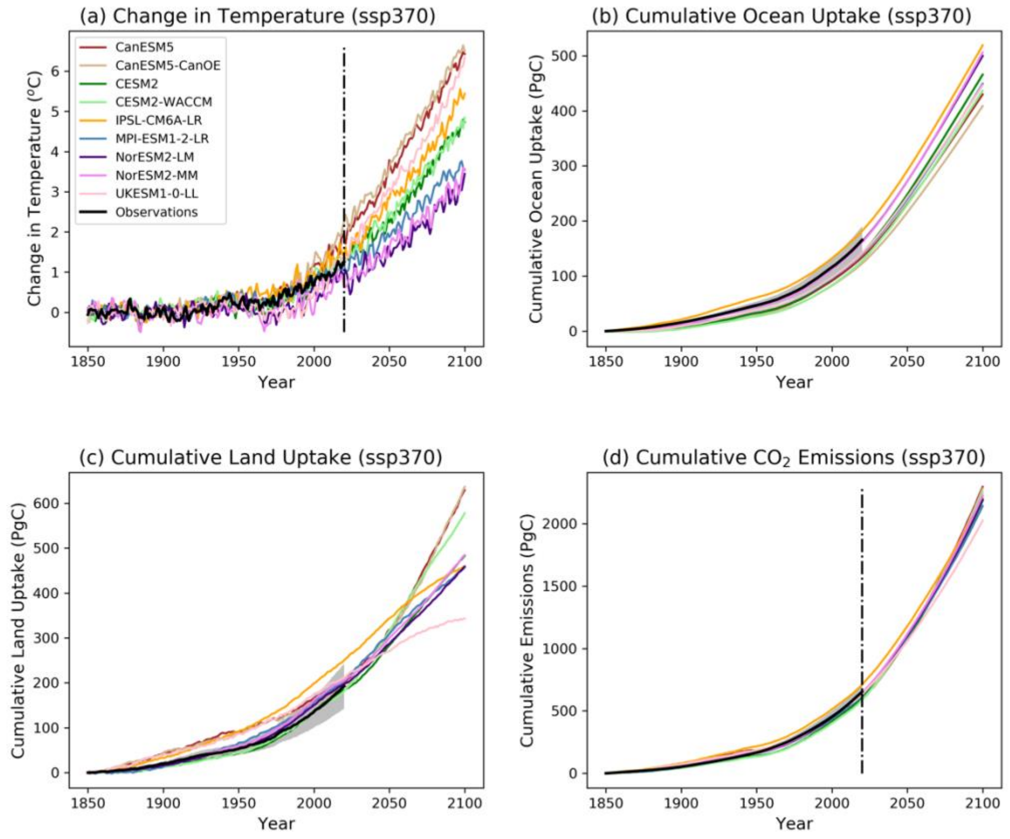

Figure S3: As figure 1 but for the SSP370 scenario, annual global mean anomalies relative to the 1850-1899 mean: (a) temperature change; (b) cumulative ocean carbon uptake; (c) cumulative land carbon uptake; (d) implied cumulative emissions (diagnosed using equation 1). Each coloured line represents a different CMIP6 Earth System Model (ESM) as identified in the key on panel (a). The thick black line in panel (a) is the observational estimate from IPCC AR6 WG1 Chapter 2. The thick black lines in panels (b)-(d) represent observational estimates from the Global Carbon Project, with grey bands showing the estimated standard error in these values (see Methods for details).

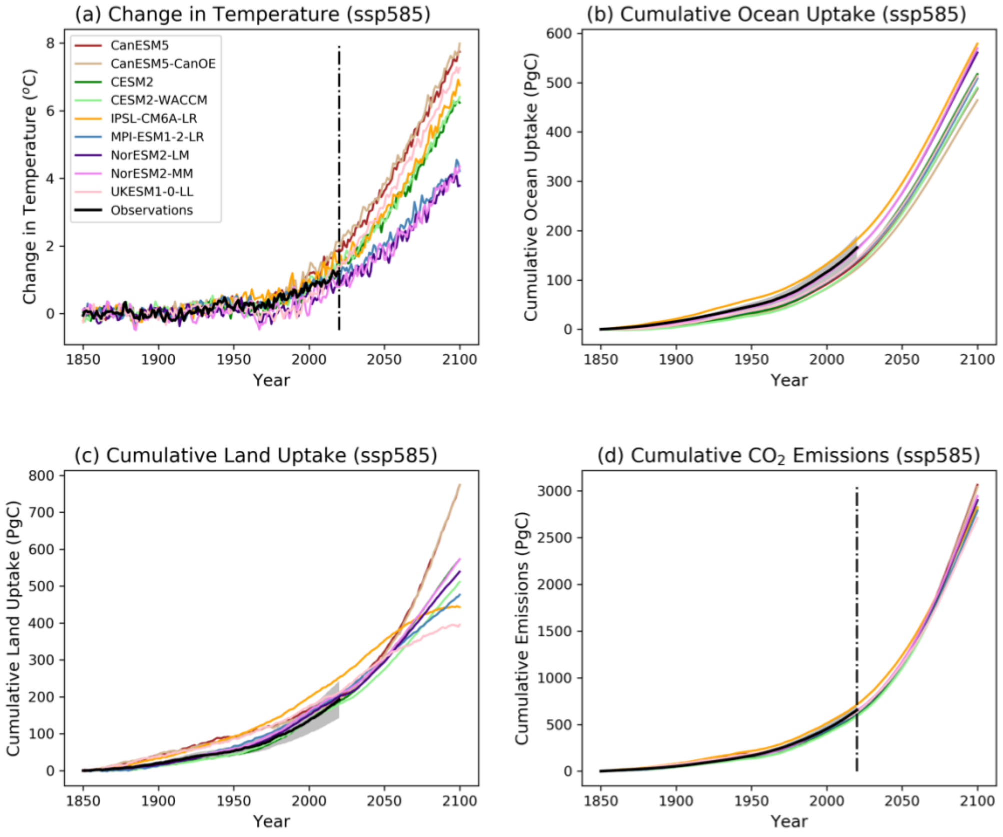

Figure S4: As figure 1 but for the SSP585 scenario, annual global mean anomalies relative to the 1850-1899 mean: (a) temperature change; (b) cumulative ocean carbon uptake; (c) cumulative land carbon uptake; (d) implied cumulative emissions (diagnosed using equation 1). Each coloured line represents a different CMIP6 Earth System Model (ESM) as identified in the key on panel (a). The thick black line in panel (a) is the observational estimate from IPCC AR6 WG1 Chapter 2. The thick black lines in panels (b)-(d) represent observational estimates from the Global Carbon Project, with grey bands showing the estimated standard error in these values (see Methods for details).

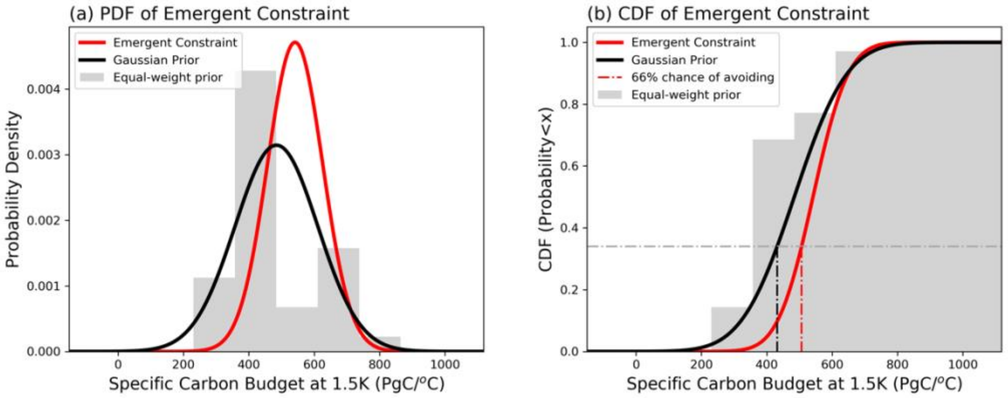

Figure S5: As figure 3 but for 1.5°C, emergent constraint on the specific carbon budget: (a) probability density function (PDF); (b) cumulative distribution function (CDF). The grey histograms show the distributions derived from the equal-weighted raw model output, and the black line is a Gaussian with the same mean and standard deviation. The thick red line shows the emergent constraint on the distribution.

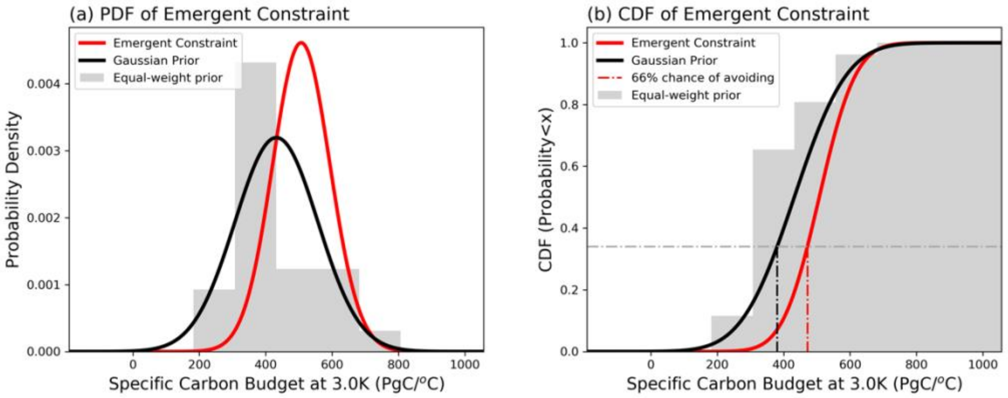

Figure S6: As figure 3 but for 3°C, emergent constraint on the specific carbon budget: (a) probability density function (PDF); (b) cumulative distribution function (CDF). The grey histograms show the distributions derived from the equal-weighted raw model output, and the black line is a Gaussian with the same mean and standard deviation. The thick red line shows the emergent constraint on the distribution.

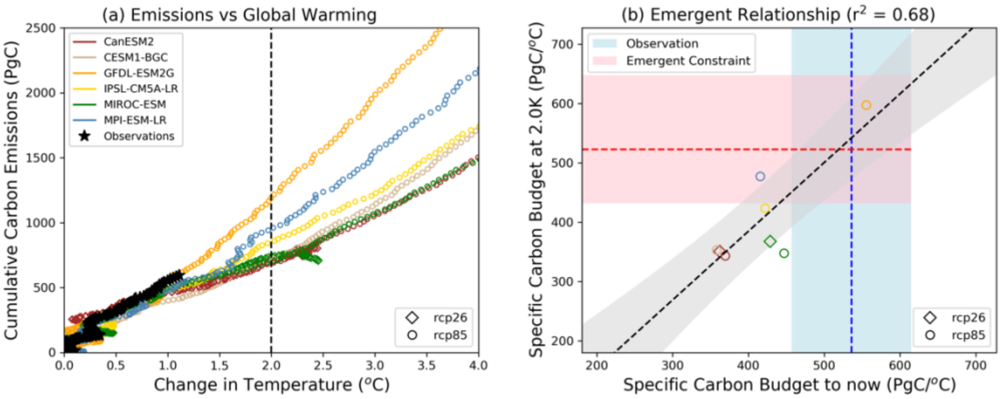

Figure S7: Relationship between cumulative emissions and global warming from CMIP5 models, for the historical simulations plus two different RCP scenarios (rcp2.6, rcp8.5). Equivalent to Figure 2 in the main manuscript, but for CMIP5 models rather than CMIP6 models. As the CMIP5 runs did not start in 1850, but instead in 1860 or 1861, the cumulative emissions are calculated relative to 1861 (rather than 1850), and global warming is calculated relative to the mean global temperature from 1861 to 1910 (rather than from 1850 to 1899).

Global Warming (K)	Carbon Budget (PgC)	
	Median	***Likely range***
1.0	588	[513, 664]
1.1	635	[555, 716]
1.2	674	[583, 766]
1.3	729	[621, 838]
1.4	772	[660, 884]
1.5	812	[691, 933]
1.6	848	[723, 974]
1.7	895	[761, 1030]
1.8	947	[797, 1097]
1.9	998	[841, 1157]
2.0	1048	[881, 1216]
2.1	1088	[914, 1263]
2.2	1143	[955, 1335]
2.3	1169	[979, 1361]
2.4	1220	[1024, 1419]
2.5	1272	[1065, 1480]
2.6	1321	[1107, 1537]
2.7	1357	[1137, 1581]
2.8	1406	[1173, 1642]
2.9	1468	[1231, 1711]
3.0	1520	[1274, 1771]
3.1	1576	[1315, 1836]
3.2	1594	[1335, 1858]
3.3	1642	[1374, 1915]
3.4	1693	[1415, 1983]
3.5	1750	[1457, 2053]
3.6	1802	[1495, 2119]
3.7	1853	[1538, 2180]
3.8	1893	[1574, 2227]
3.9	1952	[1616, 2305]
4.0	2023	[1663, 2400]

Table S1: as Table 1, but including intermediate levels of global warming and (weakly) asymmetric likely ranges.
